# Advances in mechanochemical modelling of vertebrate gastrulation

**DOI:** 10.1042/BST20240469

**Published:** 2025-07-21

**Authors:** Alex M. Plum, Mattia Serra, Cornelis J. Weijer

**Affiliations:** 1 Department of Physics, University of California San Diego, CA 92093, U.S.A.; 2 Division of Molecular Cell and Developmental Biology, School of Life Sciences, University of Dundee, Dundee DD1 5EH, U.K

**Keywords:** biological models, biophysics, computational biology, developmental biology, systems biology

## Abstract

Gastrulation is an essential process in the early embryonic development of all higher animals. During gastrulation, the three embryonic germ layers, the ectoderm, mesoderm and endoderm, form and move to their correct positions in the developing embryo. This process requires the integration of cell division, differentiation and movement of thousands of cells. These cell behaviours are coordinated through shortand long-range signalling and must involve feedback to execute gastrulation in a reproducible and robust manner. Mechanosensitive signalling pathways and processes are being uncovered, revealing that shortand long-range mechanical stresses integrate cell behaviours at the tissue and organism scale. Because the interactions between cell behaviours, signalling and feedback are complex, combining experimental and modelling approaches is necessary to elucidate the regulatory mechanisms that drive development. We highlight how recent experimental and theoretical studies provided key insights into mechanical feedback that coordinates relevant cell behaviours at the organism scale during gastrulation. We outline advances in modelling the mechanochemical processes controlling primitive streak formation in the early avian embryo and discuss future developments.

## Vertebrate gastrulation

Elucidating the mechanisms that coordinate the behaviours of large numbers of cells to sculpt and pattern the developing embryo remains one of the grand challenges of developmental biology [1–3]. Gastrulation exemplifies the importance of this coordination, as it establishes the basic embryo body plan and lays the foundation for organ formation. This process requires extensive collective cell movement to position the ectoderm, mesoderm and endoderm germ layers correctly. Tissue flow and internalization require robust and regulative coordination of cell fate and differentiation. Much of the literature has focused on the genetic control of cell behaviours, overlooking the role of mechanics. However, all embryo-scale motion is ultimately driven by mechanical forces [4]. Feedback between mechanics and biochemistry is central to understanding how such large-scale tissue movements and patterns emerge from molecular and cellular processes.

**Figure 1:**
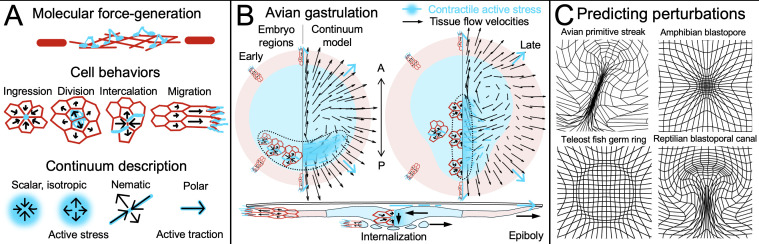
**Cell behaviours and mechanochemical modelling of chick gastrulation**. **(A**) Active force generation from cytoskeletal (top) to cell behaviours (middle) and their continuum descriptions (bottom). (**B**) Heterogeneous cell behaviours in the avian embryonic (blue) and extraembryonic (red) can be represented by continuum active stress patterns, giving rise to distinct tissue flows at different times. Mesendoderm cells (bounded by a dashed black curve) generate anisotropic active stresses (blue bars) from directed intercalation, and isotropic active stresses (blue colouring) from constriction and ingression. In early gastrulation (left), mesendoderm precursors are located in the posterior (P) embryo, and in later stages at the primitive streak along the anterior–posterior (A–P) axis (right). Cross-section (bottom) shows embryonic cells (blue) individually ingressing through the primitive streak and migrating, while extraembryonic (red) edge cells exert active traction (blue arrows) on the vitelline membrane (white), stretching the extraembryonic tissue (epiboly). (**C**) Deformed grids for perturbations to the chick mechanochemical model [7]. Deformations match those observed in experimentally manipulated chick embryos and resemble gastrulation structures in other vertebrates.

Integrating cell and tissue interactions requires short- and long-range signalling and feedback [5,6]. Short-range signalling can be achieved via diffusive signals or direct cell–cell interactions. However, the nature of long-range signalling and feedback integrating cell behaviours on the organ and organism scale remains unclear. In epithelial tissues, mechanical coupling between cells enables forces exerted locally to influence cells far away. Instead of autonomously deciding to migrate, cells can be forced to move by their neighbours, resulting in complex collective behaviours. Forces are generated primarily by cell behaviours, including division, shape change, migration, ingression and extrusion, all requiring extensive cytoskeletal remodelling, especially in the cortex (Figure 1A). Mechanical stresses associated with these behaviours can create local and global feedback between forces and tissue flows. Since many cell signalling processes and behaviours are mechanosensitive [7–14], these stresses could play a central role in coordinating cell behaviours on the embryo scale [15].

Gastrulation has been studied in several vertebrate embryos, especially frogs, fish, chick and mouse, as well as in gastrulation-mimicking stem cell models known as gastruloids [6,16]. Each model system has advantages [17]. Frog embryos are easy to maintain and manipulate, and have been the workhorse of classical embryology and the study of gastrulation for over 150 years. More recently, fish have gained popularity for their fast development, accessibility to forward and reverse genetic techniques, and their transparent embryos—ideal for imaging [18]. Avian and mouse embryos have been widely used as amniote model systems. Mouse embryos have been the primary mammalian model system because they provide access to genetic analysis crucial for dissecting signalling pathways. However, they are still challenging to culture and less amenable to experimental manipulation and direct observation of post-implantation development [19]. Avian embryos are extensively studied since embryos are easy to culture *in vitro* and readily accessible to experimental manipulation and live imaging. At the moment of egg-laying, the chick embryo contains around 50,000 cells organized in a disk-shaped embryo, consisting of an inner region, from which the embryo will develop, surrounded by an annulus of extraembryonic tissue (Figure 1B). Gastrulation stage avian embryos are essentially flat; their morphology strongly resembles human embryos, and transgenic methods are rapidly developing [20].

## Cell-scale and tissue-scale processes

Gastrulation is driven by active forces arising from energy-consuming molecular motor activity and filament polymerization. This molecular activity manifests in different cell behaviours, including *i*) cell intercalations (involving multiple cells), *ii*) active constriction, ingression or apoptosis, *iii*) cell division and *iv*) directed migration—each of which may relieve or induce stresses and strains in the tissue [21] (Figure 1A). A key challenge has been distinguishing which observed behaviours are active—cells exerting forces—and which are passive—cells responding to forces. Here, we briefly review what is known about the roles of each of these behaviours in vertebrate gastrulation. We focus on avian embryos, for which light sheet microscopy has helped uncover and quantify tissue-scale deformations and cell-scale behaviours in the whole embryo [22,23], facilitating whole-embryo modelling that does not yet exist in other vertebrate systems.

### Intercalation

Intercalation involves pairs of cells exchanging neighbours, like particles in a fluid. By bringing cells together along one axis and separating them along another axis, aligned cell intercalations can elongate tissues—a process called convergent extension [24,25]. In frogs, mesenchymal mesoderm cells intercalate before and after involution through the blastopore and epithelial cells intercalate during the forming of the neural plate. Intercalating mesenchymal cells polarize, forming active protrusions at opposite ends that pull on neighbouring cells with intracellular actin–myosin fibres. Keller and co-workers have studied this process extensively in pioneering studies using tissue explants [26,27]. In chick embryos epithelial epiblast cells intercalate [28] by shortening and remodelling cell–cell junctions through junctional actin–myosin cables [22]. This epithelial type of intercalation has also been extensively studied in *Drosophila* (fruit fly) gastrulation [29,30].

In chick mesendoderm precursors—induced in the posterior embryonic epiblast—cell shapes are initially elongated, and apical junctions perpendicular to the anterior–posterior midline exhibit elevated tension and myosin activity [31]. Tissue motion starts within a small region in this domain and then rapidly spreads outward [22], with directed intercalations converging towards the midline and inducing embryo-scale vortical tissue flows called Polonaise movements (Figure 1B). The intercalations are accompanied by the turnover of super-cellular myosin cables spanning 2–8 cell junctions that orient perpendicular to the elongating streak (Figure 1B, right). These cables appear to cause the aligned junctional contractions associated with the convergent extension, as inhibiting myosin phosphorylation and activity blocks intercalation-associated tissue flows and streak formation [22].

### Internalization

Mesoderm and endoderm precursors, in vertebrates typically arranged in a ring- or sickle-shaped domain, must be internalized during gastrulation [32,33]. In frogs and fish, mesendoderm precursors are localized in a ring-shaped domain. In frogs, these cells undergo a partial epithelial-to-mesenchyme transition (EMT) but involute as a sheet through the blastopore. In fish, mesendoderm cells are more loosely connected and ingress as a cohort through the germ ring. In avian embryos, mesendoderm precursors are formed in a posterior sickle-shaped region of the embryonic epiblast, induced by signals from the surrounding extraembryonic tissue and the underlying hypoblast [23,34].

In amniotes, mesendoderm cells undergo a complete EMT, ingress individually through the forming primitive streak and migrate away as a dense mesenchymal cohort to form various mesodermal and endodermal structures and organs.

In chick, while cell volumes remain constant, the apical cell surface areas already start to shrink before the onset of gastrulation [22], likely driven by increased myosin II apical localization and activity [7,35]. Once the streak is partially extended, cells in the mesendoderm undergo larger myosin II-driven apical contractions and complete EMT [36,37], during which they individually ingress and migrate away underneath the epiblast (Figure 1B, bottom), guided by growth factor gradients [38]. These cell ingressions pull in more mesendoderm epiblast tissue from either side of the streak until all mesendoderm precursor cells internalize.

### Cell division

Experiments in frogs and fish suggest that DNA replication and, by inference, cell divisions are not essential for gastrulation [39,40]. In avian embryos, cell divisions have been proposed to promote tissue fluidity by facilitating intercalations [41]. As in other systems, divisions in the sickle region are oriented along the axis of tissue convergence, relieving anisotropic stress [22,42]. Without division, the typical ‘Polonaise’ movements (Figure 1B) are lost after a few hours, replaced by more direct convergence towards a shorter streak [41–44].

### Epiboly

Vertebrate tissue flows are typically accompanied by the epiblast tissue expanding and thinning to cover the embryo and yolk—a process called epiboly—that ultimately encloses internalized mesoderm and endoderm germ layers. In chick, the outer extraembryonic cells attach to the overlaying vitelline membrane surrounding the egg yolk and migrate outward (Figure 1B, bottom), which increases epiblast tension [45]. This epibolic expansion is not required during streak formation, since confined embryos can still develop a streak [35], and embryos cultured on a slippery substrate in the absence of a vitelline membrane can also develop a streak [46].

## Modelling

Understanding how the behaviours of thousands of cells are robustly integrated to generate tissue flows requires mathematical modelling connecting different scales in continual feedback with experiments [47]. Models allow formalization and testing of assumptions about the mechanisms orchestrating gastrulation movements to gain insights and suggest new experiments.

Mechanochemical models of epithelial tissue dynamics have grown in recent years, incorporating mechanical forces and their chemical drivers to reproduce observed processes and patterns. Vertebrate gastrulation can be modelled at multiple scales: *i*) cell-scale models, describing individual cells or small patches of tissues; or *ii*) tissue-scale models, using a continuum approximation to describe average flows and molecular and mechanical fields associated with patches of cells. These alternative models are distinguished by the questions they can address, the types of assumptions they rely on, and their number of biophysical parameters. Several studies attempt to reconcile these different levels of description, for example, by decomposing tissue-scale deformation into the addition, subtraction, rearrangement and shape change of component cells [48–52].

### Cell-based modelling

Cell-scale models can incorporate detailed representations of cytoskeletal dynamics, highlighting the role of actin, myosin and other cytoskeletal components in generating and responding to forces [53]. These models can address how mechanochemical feedback changes individual cell shapes and stress states. Models of multicellular tissue patches include vertex models [54–59], which help understand morphogenetic motifs, such as tissue folding, invagination, elongation and convergent extension. Traditional vertex models assume that substrate friction resists cell motion, but in avian gastrulation, epiblast cells are suspended, requiring internal viscous dissipation [60]. Mechanochemical feedback could explain how cells coordinate directional intercalations, as in convergent extension. Many components in the cytoskeleton are tension-dependent, such as the unbinding rate of myosin from actin [30,61], possibly due to a catch bond mechanism [62]. Cell–cell junctions with excessive tension may accumulate more myosin, redistributed from the neighbouring cells, increasing tension on that junction in a positive feedback loop. Likewise, if a junction contracts, it may increase tension in aligned junctions of neighbouring cells, resulting in an accumulation of myosin and more contraction. This process can lead to myosin chains that drive convergent extension. Implementing this mechanism in vertex models [58] and recently developed active tension network models [56,63,64] shows how endogenous tension dynamics trigger directed intercalations and convergent extension. However, cell-based vertex models have sofar struggled to generate embryo-scale self-organizing tissue flows.

### Continuum modelling

Continuum models are best suited to investigate embryo-scale tissue flows that persist over lengths much larger than individual cell sizes, as in vertebrate gastrulation. They typically employ techniques from active matter (reviewed in [65]) and involve a relatively small number of parameters because they abstract over cell-scale details [66–71]. In the continuum, the force-generating features of cell behaviours can be captured by their symmetries, for example, through extensile or contractile isotropic stresses (in all directions), nematic stresses (with an orientation) or traction forces (with a direction) (Figure 1A, bottom).

### Forces and tissue flows

Continuum models depend on constitutive relations (stress–strain relationships) to convert active forces to flows (velocities). Some epithelial tissues can be effectively described as a two-dimensional viscous fluid generating internal active stresses. Unlike an ordinary fluid, tissues may be compressible, meaning the tissue velocity can converge and diverge. Avian gastrulation flows are highly compressible due to cell ingressions in the streak region, cell division and cell area changes. Continuum approaches recast specific cell behaviours into equivalent tissue-scale stresses and leverage simple symmetries and conservation laws to provide interpretable governing equations for tissue flows [65] (Figure 1A and B). These equations are generally efficient to simulate and allow simplified analytical predictions for embryo-scale dynamics, elucidating interactions between different model components. Continuum modelling approaches aided in understanding important processes during early development in *Drosophila*. For example, the embryo-scale viscous cytoplasm flows that drive proper nuclear positioning in the pre-cellularization *Drosophila* embryo have been successfully modelled as a composite fluid made of a thin layer of contractile actin–myosin gel, whose contractility is controlled through the cell cycle oscillator, coupled to the cytoplasm through friction [72,73]. Studies of *Drosophila* gastrulation have furthermore shown that the forces generated by the apical contraction of epithelial cell sheets are transmitted into the inner embryo regions through hydrodynamic flows of the cytoplasm, both in cellularized and in acellular embryos [74]. Other studies in *Drosophila* show that global tissue flow patterns can be predicted from experimentally measured myosin activity patterns through continuum modelling [75]. Similar continuum approaches are now being used to understand vertebrate gastrulation.

### Vertebrate gastrulation models

In vertebrates, especially in zebrafish embryos, several models have leveraged continuum approaches to describe processes related to gastrulation, such as epiboly and axis elongation [76–78]. Yet, there are few whole-embryo vertebrate gastrulation models. Existing models have taken a continuum approach and focused on avian embryos.

**Figure 2:**
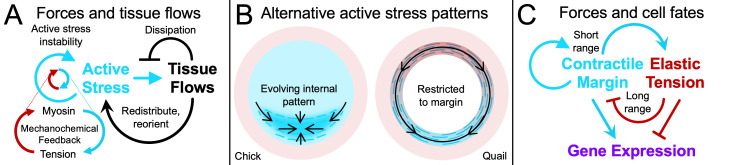
**Self-organization models for chick and quail gastrulation**. (**A**) Global integration of cell behaviours through local mechanochemical feedback and global flow coupling. Myosin contractility induces junctional tension, causing more myosin to accumulate. This mechanochemical feedback via tension-dependent myosin dynamics generates an active stress instability that increases myosin activity across the embryo over time, driving chick gastrulation flows and embryo shape change [7,35]. Tissue flows transport the cells generating active stresses, thereby redistributing and reorienting active stresses. (**B**) Alternative modelling assumptions in mechanochemical models of chick [7,35,83] and quail [43,84,85] gastrulation. (**C**) Proposed mechanochemical feedback regulating primitive streak formation in quail [84], with myosin contractility as a short-range activator and elastic tension as a long-range inhibitor. Mesendoderm gene expression is modelled as downstream of the mechanics, without feedback.

A mechanochemical model of chick gastrulation predicts the evolution of tissue flows during gastrulation using constant parameters and an initial condition for observed active stress patterns [7]. In this embryo-scale continuum model, tissue flows emerge from a balance of active and passive forces, and a boundary condition representing outward edge cell migration (Figure 1B). Active forces arise from spatial gradients in contractile myosin intensity and orientation (cables), modelled as continuous active stress fields [79–82]. Mechanochemical feedback through tension-dependent myosin dynamics produces an instability that increases activity over time [7]. Additionally, tissue flows transport and reorient myosin patterns, generating dynamic active force distributions. Passive forces reflect internal dissipation from tissue resistance to compression and viscous shear. Overall, this mechanochemical model elucidates how active forces self-organize from underlying cell behaviours (Figure 2A), and is sufficient to explain the integrated temporal development of tissue flows and actomyosin patterns during primitive streak formation (Figure 1B). A recent extension of this model further explains the embryo’s dynamic geometry—changing from circle- to pear-shaped [35]. The underlying molecular mechanisms are likely more complicated, and there is certainly additional mechanochemical heterogeneity and feedback in the tissue. However, the core principle—that active forces drive flow and that flows reshape force distributions—will also hold for more complex molecular schemes.

Forces from distinct cell behaviours shape the embryo and determine where cells separate and converge. Tissue flows over time generate cumulative deformation patterns that can be quantified via Lagrangian attractors, repellers and deforming grids (Figure 1C) [7,35,86]. To dissect the contributions of various cell behaviours to these deformation patterns, experiments in chick have been performed altering the initial shape of the mesendoderm differentiation domain from a sickle to a ring—changing the initial conditions—and interfering with EMT [83]. A ring of mesendoderm cells results in circular myosin cables and intercalations. Inhibiting EMT causes the ring to constrict and the tissue to buckle at the mesendoderm–epiblast interface. These perturbed chick embryos form structures that resemble the closing blastopore in the frog embryo. Allowing EMT and individual ingressions, the circular mesendoderm domain forms a circular primitive streak, resembling the germ ring in fish embryos. Finally, inhibiting EMT with sickle-shaped mesendoderm results in a shorter folded structure resembling the blastoporal canal in reptilian embryos. Remarkably, the mechanochemical model for chick gastrulation can capture these perturbations (Figure 1C) using the same self-organizing mechanisms (Figure 2A): simply changing the initial condition from a sickle to a ring and adjusting a parameter for myosin-driven constriction and ingression reproduces the observed tissue deformations [7].

A viscous continuum model of quail gastrulation proposes that the observed embryo-scale tissue flows could result from active stresses residing only in a ring located at the margin between the embryonic and extraembryonic tissue [43] (Figure 2B, right). It assumes that myosin contractility drives the stress gradient and requires fitting the experimentally measured tissue flow divergence throughout the embryo at every time or prescribing cumulative area changes in specific tissue regions. This model enables analysis of simplified dynamics along the ring [84]. However, it does not explain how the tensile ring is generated or why active contractility is restricted to the margin (Figure 2B). Experiments in the chick embryo have shown that apical junctional myosin evolves dynamically throughout the embryo and, after the onset of gastrulation, is dominant in the streak region and minimal at the embryo-extraembryonic margin (Figure 1B [7,35,83]). A recent viscoelastic extension of the tensile ring model further predicts velocity divergence fields by incorporating evolving elastic stress. This still requires prescribing ingression dynamics in the streak region and contractility dynamics on the ring [85] to obtain quail gastrulation flows.

A question in avians is why the mechanically coupled embryonic and extraembryonic areas do not both stretch and thin under epiboly. The quail model proposes that a tensile ring shields the embryonic region from epiboly-induced stretching. In contrast, the chick model attributes the lack of stretching to internally distributed myosin contractility throughout the embryonic region (Figure 2B) [35]. Distinguishing whether embryonic cells collectively resist stretching through contractility or are shielded calls for comparison of absolute cell area dynamics across the embryo and margin in perturbations, along with viscoelastic models of cell area dynamics that incorporate cell divisions and ingressions.

### Forces and cell fates

Mesendoderm precursor cells are initially induced by conserved molecular signals from the future endoderm and extraembryonic tissues, involving members of the Bone Morphogenetic Protein (BMP), Transforming Growth Factor Beta (TGF*β*), and Wnt families [87]. These cells are the first to ingress in the forming streak. However, as cells ingress, new mesendoderm cells must be recruited non-cell-autonomously beyond the initial pattern. Whether cells switch on mesendoderm genes when they move near the streak and which signals control those changes are open questions. Mechanical stresses associated with ingression in the streak could be involved in controlling mesoderm or endoderm gene expression in these cells, which will be important to explore in further experiments and modelling. In line with increasing evidence for mechanochemical control of gene expression during early development [88], a recent study of quail embryos proposed that mesendoderm gene expression depends on cumulative active tissue contraction. Extending the tensile ring model (Figure 2B), this study proposes that elastic tension is a long-range inhibitor of mesendoderm differentiation, and local contractility a short-range activator, forming a self-organizing patterning system to prevent the formation of more than one streak and explain additional (ectopic) streaks observed in experimental perturbations [84] (Figure 2C). This hypothesis is supported by experimental observations showing that streak formation was inhibited where contractility was inhibited. Whether these gene expression changes are purely downstream (Figure 2C) or also feedback on forces remains unclear. Additional mechanisms—such as morphogen-mediated interactions within the contractile region [89], potentially influenced by deformation [90,91], or interplay with other biochemical regulators of streak formation [92,93]—may also be important. This rapidly emerging research area requires further experimental and model-based validation.

**Perspectives**

- *Importance of the field*: Multicellular morphogenetic processes, such as gastrulation—the process that shapes the developing embryo—are driven by dynamic, active forces operating across molecular, cellular and tissue scales. Understanding how these forces are generated and how they feed back on biochemical reactions and emergent cellular and tissue behaviours requires an integrative approach that combines experimentation with modelling. This comprehensive understanding will advance our knowledge of development and has implications for medicine and biological engineering.
- *Summary of current thinking*: Recent advances have identified key mechanochemical feedback mechanisms driving epiblast tissue flows during gastrulation in vertebrates. Whole-embryo modelling efforts have focused on primitive streak formation in essentially flat avian embryos under normal and perturbed conditions.
- *Future directions*: In the transition to later developmental stages, the primitive streak buckles, the forming neural plate folds to form the brain, and gene expression programmes further diversify. Understanding these processes will require a combination of cell-based and continuum models in three dimensions, incorporating new mechanochemical signalling mechanisms, cell–cell signalling networks and regulatory switches for cell behaviours. These advances will enable the first comprehensive understanding of morphogenesis and differentiation to elucidate how embryos acquire their form and function.

## Abbreviations

BMP: Bone Morphogenetic Protein
EMT: epithelial-to-mesenchyme transition
TGFβ: Transforming Growth Factor Beta
WNT: Wingless/Integrated family of signalling molecules
